# Calcium Charge and Release of Conventional Glass-Ionomer Cement Containing Nanoporous Silica

**DOI:** 10.3390/ma11081295

**Published:** 2018-07-27

**Authors:** Koichi Nakamura, Shigeaki Abe, Hajime Minamikawa, Yasutaka Yawaka

**Affiliations:** 1 Department of Dentistry for Children and Disabled Person, Graduate School of Dental Medicine, Hokkaido University, Kita 13 Nishi 7, Kita-ku, Sapporo 060-8586, Hokkaido, Japan; yawaka@den.hokudai.ac.jp; 2 Department of Biomaterials and Bioengineering, Graduate School of Dental Medicine, Hokkaido University, Sapporo 060-8586, Hokkaido, Japan; sabe@den.hokudai.ac.jp; 3 Department of Dentistry for Molecular Cell Pharmacology, Graduate School of Dental Medicine, Hokkaido University, Sapporo 060-8586, Hokkaido, Japan; minami@den.hokudai.ac.jp

**Keywords:** nanoporous silica, glass-ionomer cement, calcium

## Abstract

The aim of this study was to evaluate calcium charge and release of conventional glass-ionomer cement (GIC) containing nanoporous silica (NPS). Experimental specimens were divided into two groups: the control (GIC containing no NPS) and GIC-NPS (GIC containing 10 wt % NPS). The specimens were immersed in calcium chloride solutions of 5 wt % calcium concentration for 24 h at 37 °C, whereupon the calcium ion release of the specimens was measured. The calcium ion release behavior of GIC-NPS after immersion in the calcium solution was significantly greater than that of the control. Scanning electron microscopy and electron-dispersive X-ray spectroscopy results indicated that calcium penetrated inside the GIC-NPS specimen, while the calcium was primarily localized on the surface of the control specimen. It was demonstrated that NPS markedly improved the calcium charge and release property of GIC.

## 1. Introduction

The overall incidence of dental caries has gradually decreased, but it still occurs quite frequently [1]. In the case of small dental carious lesions, the tooth is typically restored by a coronal restoration material, which often requires a retreatment owing to circumstances such as the progress of the dental caries around the restoration or the dental restoration falling out [2,3]. The main cause of coronal restoration treatment failure is secondary caries, which requires coronal restoration or pulp treatment [4,5]. To avoid tooth loss, therefore, it is necessary to prevent secondary caries, for which fluoride takes an important role [6,7]. Fluoride ions are included in dental items such as toothpaste and glass-ionomer cement (GIC) [8,9], and GIC is often used to prevent secondary caries in children [10,11]. GIC is widely used in dentistry such as the luting cement [12], temporary restoration [13], and adhering orthodontic band [14]. The GIC supplemented with TiO_2_ nanoparticles also have the effect of antibacterial properties [15].

Calcium is an important component of the tooth. It is essential for the tooth mineralization that the calcium ion exists around the tooth. As one of the methods to supply calcium for teeth, some studies proposed the addition of casein phosphopeptide amorphous calcium phosphate (CPP-ACP) to GIC [16,17,18]. Addition of CPP-ACP to GIC increased the release of calcium ions. However, CPP-ACP is an ingredient derived from milk, and clinicians should consider potential side effects from ingestion of casein derivative protein in people with immunoglobulin E allergies to milk proteins [19].

Nanoporous silica (NPS) has a structure that possesses a uniform pore size of about 1 nm with a large specific surface area and is attractive in applications such as a sustained drug release carrier and catalyst support [20]. It is believed that NPS can adsorb formaldehyde gas and methylene blue pigments, and it has been confirmed that one of the properties of NPS is its ability to adsorb various ions and substances [21,22,23]. However, there are few studies on the application of NPS in dentistry.

The purpose of this study was to determine whether conventional GIC containing NPS was able to charge and release calcium ions. Furthermore, the cross-section of the GIC-NPS specimen was observed by scanning electron microscopy (SEM) and the compressive strength was measured. Our null hypotheses were that GIC containing the NPS did not charge and release calcium ions, and that there was no difference in the compressive strength of the GIC control and the GIC-NPS.

## 2. Materials and Methods

### 2.1. NPS Synthesis

The NPS was synthesized in accordance with the protocol described by Tagaya et al. [20]. In this process, 1.37 mmol of cetyltrimethylammonium bromide (CTAB: Wako, Osaka, Japan) was added to 120 mL of distilled water, to which 1.75 mL of 2.0 M sodium hydroxide (Wako) solution was added. The mixture was stirred for 30 min at 80 °C, whereupon 12.4 mmol of tetraethoxysilane (Wako) was added and the mixture stirred for 2 h at 80 °C. The resulting suspension was filtered and dried and, to remove the CTAB, the obtained particles were calcined at 550 °C for 4 h. The obtained white particles were subsequently observed using an SEM (S-4800, HITACHI, Tokyo, Japan) and a transmission electron microscope (TEM: JEM-2010, JEOL, Tokyo, Japan).

### 2.2. Preparation of Test Specimens

The obtained NPS was added to the powder component of the GIC (GC Fuji II, GC, Tokyo, Japan; lot 1606141) at 10 wt % concentration, whereupon the test specimens were prepared by mixing the powder component (with or without NPS) and the liquid component of the GIC (GC Fuji II, GC, Tokyo, Japan; lot 1607041) in accordance with the manufacturer specifications. The specimens were set in two patterns, where the first was 10 mm in diameter and 1 mm in height for the calcium ion release measurement and the SEM and electron-dispersive X-ray spectroscopy analysis, and the second was 4 mm in diameter and 6 mm in height for the compressive testing. The experimental specimens were divided into two groups comprising the control (no NPS) and the GIC-NPS (containing 10 wt % NPS). The specimens were wet ground via hand lapping using P400 grit silicon carbide abrasive papers (SANKYO, Saitama, Japan).

### 2.3. Calcium Charge and Release

Three 10 mm-diameter, 1 mm-height specimens were immersed in a calcium chloride solution with a 5 wt % calcium concentration (calcium 150 mg/3mL) for 24 h at 37 °C. After removal from the calcium solution, the specimens were rinsed with distilled water and subsequently immersed in 5 mL of distilled water at 37 °C for 7 d. The distilled water was changed every day and was further analyzed daily to determine the calcium ion concentration in the water. N = 6 samples (18 specimens) per group were used. The calcium ion concentration was measured using inductively coupled plasma atomic emission spectroscopy (ICPE-9000, SHIMADZU, Tokyo, Japan). The total weights of the calcium releasing from the specimens were calculated from the calcium concentration. The preparation of specimens was carried out according to Bando’s conditions [24], but slightly modified.

### 2.4. Scanning Electron Microscopy (SEM) and Energy-Dispersive X-ray Spectroscopy (EDS)

Specimens containing calcium were analyzed using SEM and EDS (Genesis, EDAX Japan, Tokyo, Japan). The specimens were immersed in the 5 wt % calcium solution and dried. The specimens were cut perpendicularly, then the specimen surface and cross-section were observed after carbon coating.

### 2.5. Compressive Strength Test

The 4 mm-diameter, 6 mm-height test specimens were placed in a universal testing machine (Model 4204, Instron, Canton, OH, USA) with a cross-head speed of 1.0 mm/min. N = 12 specimens per group were used for this test. This procedure has been described in detail elsewhere [25].

### 2.6. Statistical Analysis

Statistical analysis was performed using IBM SPSS Statistics Version 21 (IBM Japan, Tokyo, Japan), and the results were analyzed statistically using the Mann-Whitney U test. The level of significance was set at *p* < 0.05.

## 3. Results

### 3.1. Morphological Characteristics of NPS

**Figure 1:**
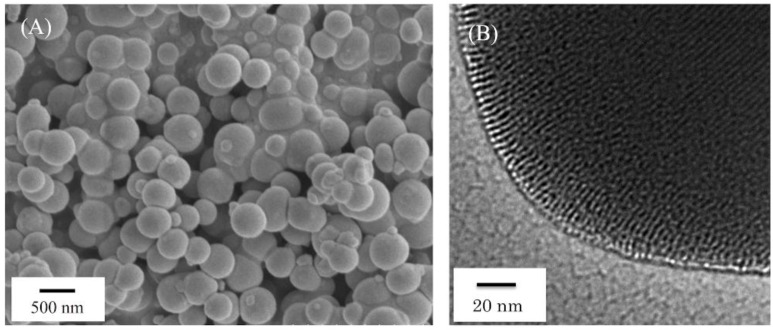
Typical (**A**) SEM and (**B**) TEM images of nanoporous silica particles. The nanoporous silica particles showed sizes approximately 200–300 nm in diameter (**A**), exhibiting pores a few nanometers in diameter (**B**).

The SEM and TEM images of NPS particles are shown in Figure 1. The NPS particles were spherical and approximately 200–300 nm in diameter, exhibiting pores a few nanometers in diameter.

### 3.2. Calcium Charge and Release

**Figure 2:**
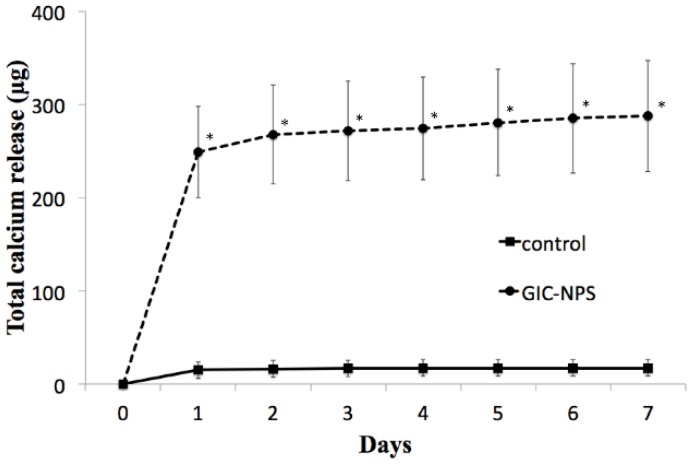
Time-profile of calcium release from the specimens after immersion in 5 wt % calcium chloride solution. Significance was determined using the Mann-Whitney U test (*p* < 0.05).

Figure 2 shows the time-profile of the release of calcium from the specimens after immersion in the 5 wt % calcium chloride solution. The calcium release behavior of GIC-NPS was significantly greater than that of the control (*p* < 0.05).

### 3.3. EDS Analysis

**Figure 3:**
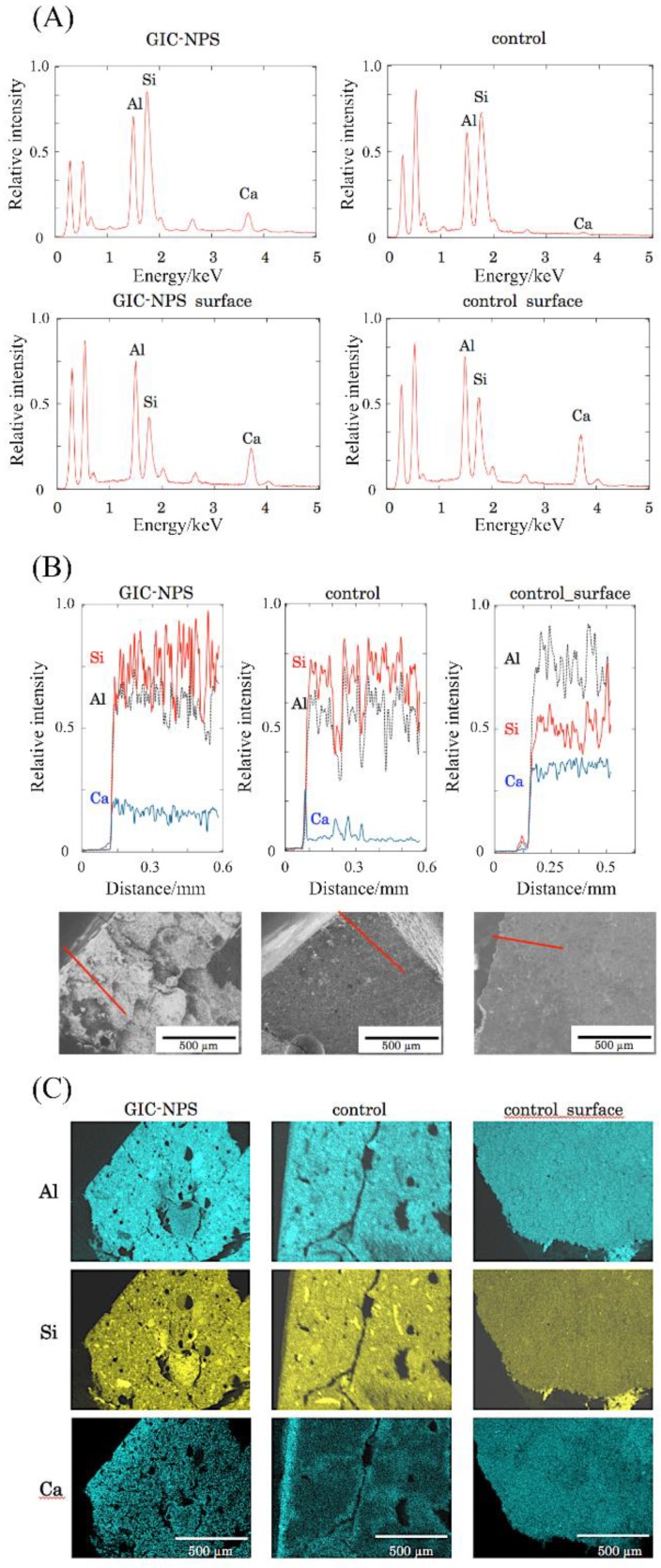
(**A**) Typical EDS spectra from the cross-section (upper) and surface (lower) of the GIC-NPS (left) and control (right) specimens, displaying the constitutive elements. (**B**) SEM images (lower) and EDS line analysis (upper) for the GIC-NPS cross-section (left), control cross-section (center), and control surface (right). (**C**) EDS element mapping of the Al (upper), Si (center), and Ca (lower) in the GIC-NPS cross-section (left), control cross-section (center), and control surface (right).

Figure 3A–C show the results of SEM and EDS for the surface and the cross-section of the specimens. As shown in Figure 3A, calcium was detected on the surfaces of both the control and GIC-NPS specimens; however, the specimen cross-sections exhibited significant amounts of calcium only for the GIC-NPS specimen. Although calcium was localized primarily on the surface of the control specimen, it was observed on the surface and throughout the inside of the GIC-NPS specimen (Figure 3B,C). In the surface of the control specimen (Figure 3B right and 3C right), calcium was distributed uniformly.

### 3.4. Compressive Strength

The compressive strength of the control and GIC-NPS were 111.37 ± 28.75 MPa and 100.32 ± 20.73 MPa (Mean ± SD, N = 12). There was no significant difference between the compressive strength of the control and GIC-NPS (*p* = 0.319).

## 4. Discussion

In the present study, the amount of the calcium release from the glass-ionomer cement containing nanoporous silica (GIC-NPS) specimen was determined. The results suggested the existence of an NPS-induced calcium ion charge and release property of the GIC-NPS. Even when a concentration of 10 wt % NPS was included in the GIC, the obtained specimens exhibited compressive strength, comparable to that of conventional GIC without NPS. It has been suggested that the possibility exists of NPS being used in dental materials as a calcium source, and it is therefore important that NPS did not reduce significantly the strength properties of the dental materials in the present study. However, other properties (solubility, adhesive strength, etc.) of GIC-NPS still have not been clarified, and so it is necessary for those concerned to hold further studies.

The nanopores in the NPS were approximately the size through which a calcium ion can pass [26]. The EDS images of the GIC-NPS specimens indicated that calcium ions penetrated into the GIC-NPS after immersion in the calcium solution. The NPS is well known as an adsorbent particle, and calcium might be adsorbed in the NPS pores in the same way other substances are adsorbed. It was not confirmed, however, which component of the NPS combined with the calcium, but this should be the subject of future research.

There were 150 mg of calcium in the immersed solution; nevertheless, the total amounts of the calcium released from specimen of control and GIC-NPS were 17.21 ± 8.66 μg and 287.71 ± 56.60 μg, respectively. Almost all of the calcium remained in the immersed solution or was washed away by rinsing with water. The percentage of total calcium released from the specimen in GIC-NPS on the first day, the second day, and the third day were 86.6%, 6.5%, and 1.5%, respectively. In contrast, those percentages for the control on the first day, the second day, and the third day were 87.7%, 6.6%, and 3.3%, respectively. Though the total amount of calcium was different for both, the percentage of calcium released had a similar tendency in both.

In this study, NPS was found to not only adsorb calcium ion but also to release adsorbed calcium ions when placed in an aqueous medium. The GIC-NPS could be expected to function as a source of calcium, and it was suggested that it could be useful to facilitate remineralization. Furthermore, the GIC used in this study was a fluoride-releasing material; thus, GIC-NPS may be useful for producing fluoroapatite by allowing the coexistence of calcium and fluoride ions [27]. This is significant because fluoroapatite possesses a higher acid resistance than hydroxyapatite, the main component of teeth. The amount of fluoride ions released from the GIC depends on the fluoroaluminosilicate glass composition included in the powder [28]. In this study, the inclusion of NPS decreases the relative amount of the GIC powder, so it is likely that the amount of fluoride ions released may be slightly decreased. This reduction may be negligible with a 10 wt % concentration of NPS, however, because of the amount of fluoride ions released from the GIC [29]. Furthermore, the specimens were immersed in calcium chloride solution in this study. Previous research has indicated that the surface hardness of GIC increases with immersion in the calcium chloride solution [30]. The surface property of GIC may have changed by immersion of the solution.

The SEM/EDS analysis indicated that calcium penetrated to the interior of the GIC-NPS specimen. After immersion in the calcium solution, calcium was distributed homogenously on the surfaces of the control specimen similar to the distribution of aluminum and silicon, which are the major constituents of GIC (Figure 3C, right column). However, the distribution behavior in the depth direction was clearly different in the two specimens. In the case of the control, calcium was localized on the surface of specimen (Figure 3C, middle column), while calcium was detected even inside of the GIC-NPS (Figure 3C, left column). The highest concentration of calcium was determined to be on the surface of the GIC-NPS specimen, similar to that found for the control specimen. The EDS mapping suggests that the calcium concentration slightly decreased as a function of depth but persisted throughout the interior of the GIC-NPS specimen.

The EDS spectral line analysis (Figure 3B) also revealed that the calcium concentration profile of the GIC-NPS specimen was clearly different than that of the control specimen. The latter exhibited a sharp peak only around the specimen surface, while the former exhibited a calcium concentration that slightly decreased in a path from the surface to a depth of 0.1 mm, then remained approximately constant up to 0.5 mm of depth.

Nanoporous silica is often applied as a film on the material surface and is rarely contained inside the material [31]. Nevertheless, it was found herein that NPS was capable of the charge and release of calcium ions through the matrix even when the NPS was incorporated throughout the material.

Previous studies of powdered inorganic additives have shown the compressive strength increase and decrease [25,32,33]. In this study, compressive strength has slightly decreased. It may be due to interference of the NPS with the normal GIC reaction. By increasing of NPS component, compressive strength might significantly decrease. However, under the conditions of the present study, there were no significant differences between the two groups.

The appearance of secondary caries requires a certain period time. In the present study, however, the calcium charge and release properties of GIC-NPS were observed over only a week. If it is a repeated charge and release of the calcium, secondary caries were prevented in the long term. To confirm this presumption, further study is needed. We also measured the amount of calcium charge and release; the study of the preventing effect for secondary caries may be needed in the model similar to the oral cavity.

In recent years, many studies of bioactive materials have been conducted [34,35,36]. The results of this study may be useful to the development of biomaterials. Firstly, the results herein may be extended to the development of remineralization-inducing materials by the uptake of phosphate ions, which are essential for remineralization, e.g., pit and fissure sealants and restoration materials. Also, this study may inform the drug delivery system by the uptake of antibacterial agents such as Cetylpyridinium Chloride (CPC). Furthermore, the NPS used in this study possesses a negative charge, and therefore cannot adsorb a negatively-charged fluoride ion. However, the NPS may be enabled to adsorb a fluoride ion by also causing it to retain a positive electric charge. This would give the ability of fluoride ion release to the material without also requiring a composite resin that exhibits fluoride ion release characteristics. We believe that it is necessary to study these possibilities in future work.

## 5. Conclusions

We demonstrated the capacity of GIC-NPS for calcium ion charge/release and contrasted it with that of conventional GIC. The presence of NPS was found to markedly improve the calcium ion charge/release property of GIC. Even for NPS concentrations up to 10 wt % in the GIC, the compressive strength of the GIC was not changed significantly.

## Supplementary Materials

The following are available online at http://www.mdpi.com/1996-1944/11/8/1295/s1, Table S1: Total calcium release, Table S2: Compressive strength.
