# Supplementary material for: Calcium Charge and Release of Conventional Glass-Ionomer Cement Containing Nanoporous Silica
Source: Materials (Basel). 2018 Jul 27;11(8):1295. doi: 10.3390/ma11081295 (PMC6117706; doi:10.3390/ma11081295)
Supplement: Supplementary file 1 [file materials-11-01295-s001.pdf]

Supplemental

**Table 1.** Total calcium release

Total calcium release (μg)

| | 1d | 2d | 3d | 4d | 5d | 6d | 7d |
|---|---|---|---|---|---|---|---|
| control 1 | 2.84 | 3.91 | 4.67 | 5.15 | 5.15 | 5.15 | 5.15 |
| control 2 | 11.00 | 12.78 | 14.22 | 14.51 | 14.51 | 14.51 | 14.51 |
| control 3 | 16.60 | 16.60 | 16.60 | 16.60 | 16.60 | 16.60 | 16.60 |
| control 4 | 30.40 | 32.10 | 32.10 | 32.10 | 32.10 | 32.10 | 32.10 |
| control 5 | 14.50 | 15.50 | 16.15 | 17.80 | 17.80 | 17.80 | 17.80 |
| control 6 | 15.20 | 16.40 | 16.95 | 17.10 | 17.10 | 17.10 | 17.10 |
| | | | | | | | |
| GIC-NPS 1 | 307.05 | 316.29 | 318.09 | 318.59 | 319.77 | 328.18 | 332.06 |
| GIC-NPS 2 | 223.15 | 230.26 | 230.80 | 230.80 | 232.21 | 237.22 | 239.33 |
| GIC-NPS 3 | 287.00 | 315.40 | 318.55 | 323.66 | 334.16 | 337.06 | 338.76 |
| GIC-NPS 4 | 169.00 | 183.80 | 189.40 | 190.60 | 195.44 | 196.94 | 197.44 |
| GIC-NPS 5 | 261.60 | 299.00 | 310.60 | 317.30 | 327.50 | 335.75 | 339.00 |
| GIC-NPS 6 | 246.80 | 261.25 | 263.75 | 265.70 | 274.45 | 274.95 | 279.70 |

.

**Table 2.** Compressive strength

Compressive strength

| | MPa |
|---|---|
| Control 1 | 117.60 |
| Control 2 | 121.42 |
| Control 3 | 105.89 |
| Control 4 | 95.70 |
| Control 5 | 150.08 |
| Control 6 | 109.87 |
| Control 7 | 155.65 |
| Control 8 | 147.37 |
| Control 9 | 85.67 |
| Control 10 | 90.92 |
| Control 11 | 95.86 |
| Control 12 | 60.35 |
| GIC-NPS 1 | 137.74 |
| GIC-NPS 2 | 118.07 |
| GIC-NPS 3 | 94.43 |
| GIC- NPS 4 | 104.70 |
| GIC-NPS 5 | 100.64 |
| GIC-NPS 6 | 105.57 |
| GIC- NPS 7 | 118.55 |
| GIC-NPS 8 | 110.03 |
| GIC-NPS 9 | 85.59 |
| GIC-NPS 10 | 89.73 |
| GIC-NPS 11 | 57.96 |
| GIC-NPS 12 | 80.81 |

.
